# Loss of association between HbA1c and vascular disease in older adults with type 1 diabetes

**DOI:** 10.1371/journal.pone.0234319

**Published:** 2020-06-15

**Authors:** HaEun Ji, Ian Godsland, Nick S. Oliver, Neil E. Hill

**Affiliations:** 1 Imperial College Healthcare NHS Trust, London, United Kingdom; 2 Division of Diabetes, Department of Metabolism, Endocrinology and Metabolism, Digestion and Reproduction, Faculty of Medicine, Imperial College London, London, United Kingdom; Swansea University, UNITED KINGDOM

## Abstract

**Aims:**

Robust evidence supports intensive glucose control in those with recently diagnosed type 1 diabetes to reduce the risk of developing micro- and macrovascular complications. Data to support longitudinal glycaemic targets is lacking. We aimed to explore if longer duration of diabetes and greater age might reduce the impact of glycaemia on the risk of vascular complications.

**Research and design methods:**

Data for adults age 20 years or more, was extracted from a clinical database of people with type 1 diabetes cared for at a London teaching hospital. The presence or absence of micro- and macro-vascular complications was recorded. Multivariable logistic regression analysis was performed using HbA1c as independent variable, diabetes duration and age as continuous variable and obesity, hypertension, hypercholesterolaemia, low HDL cholesterol and hypertriglyceridaemia as categorical variables.

**Results:**

Data from 495 patients was used. HbA1c above 60 mmol/mol (7.6%) was associated with increased microvascular complications in patients aged 20–44 years, independent of age and duration of diabetes. In older people with T1DM duration of diabetes was the major risk factor.

**Conclusions:**

Our study suggests that increased age and greater duration of diabetes reduce the impact of glycaemia on the risk of vascular complications. Intensive blood glucose management in patients aged ≥45 years may have limited benefits in terms of reducing the risk of complications although this does not dismiss the benefits of good glycaemic control in older people with T1DM.

## Introduction

All subtypes of diabetes are associated with an increased risk of long-term micro- and macrovascular complications, which may determine future quality of life and mortality [1]. The benefits of early intensive glycaemic control in newly-diagnosed type 1 diabetes mellitus (T1DM) are well-established [2] and have been shown to persist [3].

To minimise the risk of complications relating to hyperglycaemia, the National Institute for Health and Care Excellence (NICE) suggest a target HbA1c of 48 mmol/mol (6.5%) or lower where possible [4,5]. There is recognition that glycaemic targets may need to be relaxed in older, frail patients but evidence to support this is lacking [6].

There is also evidence to suggest that clinically meaningful improvements in blood glucose levels rarely occur after 5 years from diagnosis [7,8]. Collectively, these data argue for early and intensive glycaemic control in people with T1DM but data to inform longitudinal glycaemic targets in T1DM are lacking.

In this cross-sectional, preliminary analysis, using available clinical database information we aim to address the hypothesis that there may be a weaker or no relationship between HbA1c and vascular risk in older people with Type 1 diabetes.

## Methods

Data were derived from a database of information recorded for people with T1DM who attended the adult diabetes clinic at the Charing Cross and Hammersmith Hospitals, London, UK. All data was anonymised before being accessed and analysed. The patient’s medical records were accessed between February and April 2016. The earliest and latest review dates in the data file are 7 August 2003 and 18 March 2016. The medical records were accessed from Imperial College Healthcare NHS Trust. Consent was not sought because the data was analysed anonymously. For the present analysis, clinical information, as recorded by clinicians at each participant’s review, was considered for the most recent review at the time of data extraction. Data was restricted to the adult range of 20 years of age or more, during which increasing prevalence of vascular disease with age might be expected. Macro- (ischaemic heart disease, peripheral vascular disease, strokes) and microvascular (retinopathy, nephropathy, neuropathy) complications, were classified as ‘present’ or ‘absent’, based on most recent clinical status, which was recorded as free text string information for each participant. Diagnosis of retinopathy was based on results from the national screening programme, nephropathy on laboratory normal ranges for urine microalbumin:creatinine ratio, and neuropathy on clinical examination findings. Data extracted also included: date of birth, review date, date of diabetes diagnosis, resting blood pressure (BP), body mass index (BMI), HbA1c, serum total, low density lipoprotein (LDL) and high density lipoprotein (HDL) cholesterol and serum triglycerides and whether or not anti-hypertensive or anti-lipid medication were prescribed.

Ranges of age for analysis of associations between glycaemia and vascular disease derived from tertiles of age at review to provide ‘young adult’, ‘middle-age’ and ‘elderly’ groupings. Tertile age ranges were: 20.15–44.12 years (n = 167) 44.39–59.12 years (n = 167) and 59.13–89.54 years (n = 166). Based on these ranges, the integer age ranges: ≤44 (n = 173); 45–59 (n = 177) and ≥60 (n = 145) years were chosen for primary analyses. In secondary analyses to enable, with exactly equal-sized groupings, confirmation of associations distinguished in the primary analysis, associations between glycaemia and vascular disease in exact tertile ranges for the groups being investigated were also explored. Complications present in each participant were categorised as: 0, 1, or ≥2 microvascular complications and 0 or ≥1 macrovascular complications.

Statistical analyses (Mann-Whitney and logistic regression) used Prism 7 for Windows (GraphPad, San Diego, California, USA) or STATA 13 for Windows (Stata, College Station, TX, USA). Odds ratios for categories of vascular disease were explored in multivariable logistic regression analysis in three groups of participants: 1) those with a single microvascular disease or no microvascular disease; 2) those with two or more microvascular diseases or no microvascular disease; and 3) those with any macrovascular disease or no macrovascular disease. In these analyses, the primary independent variable was HbA1c concentration or HbA1c category. Other independent variables included in the analysis were: diabetes duration and age (within each age category) as continuous variable and obesity (BMI ≥30kg/m^2^), hypertension (≥140/90 mmHg, or on blood pressure lowering medication), hypercholesterolaemia (LDL ≥2.5 mmol/L, or on lipid lowering medication), low HDL cholesterol (<1.03 mmol/L) and hypertriglyceridaemia (≥1.7 mmol/L) as categorical variables. A significance level of p<0.05 was adopted for interpretation.

## Results

The analysis included 495 patients with complete data. Demographic data and group characteristics with vascular disease prevalence in each age category are shown in Table 1.

**Table 1:** Individual characteristics and prevalence of micro- and macrovascular complications in 3 age ranges for 495 people with T1DM reviewed between 2003 to 2016 at Charing Cross and Hammersmith Hospitals, London, UK. For continuous data, median (range) and for categorical % (n) are shown. For definitions of categorical variables, see Methods.

	Age group (years)		
	20–44 (n = 173)	45–59 (n = 177)	≥60 (n = 145)
Age at review (years)	36 (20–44)	53 (45–59)	67 (60–89)
Age at diagnosis (years)	14 (0.3–40)	20 (0.9–49)	30 (7–82)
Duration of diabetes (years)	21 (0.25–41)	32 (4–54)	37 (6–63)
Female (%)	60 (104)	50 (89)	44 (64)
White ethnicity (%)	80 (138)	81 (144)	90 (130)
HbA1c (mmol/mol)	65.0 (27.9–198.4)	66.1 (32.2–126.2)	61.7 (30.1–106.6)
HbA1c (%)	8.1 (4.7–20.3)	8.2 (5.1–13.7)	7.8 (4.9–11.9)
Obesity (%)	12 (21)	26 (46)	17 (25)
Hypertension (%)	43 (74)	77 (137)	86 (124)
Hypercholesterolaemia (%)	78 (138)	93 (165)	96 (139)
Low HDL cholesterol (%)	8 (14)	14 (24)	12 (18)
Hypertriglyceridaemia (%)	43 (74)	86 (152)	92 (134)
**Microvascular complications**	46 (79)	63 (112)	69 (100)
retinopathy*	92 (73)	92 (103)	82 (82)
nephropathy*	39 (31)	32 (36)	40 (40)
neuropathy*	14 (11)	20 (22)	28 (28)
**Macrovascular complications**	2 (3)	17 (30)	34 (49)
ischaemic heart disease*	33 (1)	67 (20)	65 (32)
peripheral vascular disease*	100 (3)	40 (12)	43 (21)
stroke*	0	13 (4)	18 (9)
**Both micro- and macrovascular**	2 (3)	15 (26)	23 (34)
**Two or more microvascular**	19 (32)	25 (44)	31 (45)
**No complications**	54 (94)	34 (61)	22 (32)

* as a percentage of those with micro- or macrovascular complications

In multivariable logistic regression analysis, the odds ratio for each of the vascular disease categories relative to no vascular disease was independently increased by duration of diabetes in all age groups except macrovascular disease in age group 1, in which, with only 3 cases, odds could not be estimated (Table 2). Odds ratios ranged between 1.04 and 1.12 (p = 0.01 to <0.001), depending on vascular disease category and age group. In addition, in age group 1 (20–44 years) the odds ratio for both 1 microvascular disease and ≥2 microvascular diseases was independently increased by hypertension (OR 2.3, p = 0.05 and 11.3, p<0.001, respectively) and the odds ratio for ≥2 microvascular diseases was independently increased by increasing HbA1c concentration (OR 1.03, p = 0.004). Also in addition to duration of diabetes, the odds ratio for ≥2 microvascular diseases was incidentally increased in age group 2 by ‘high LDL cholesterol or use of an lipid lowering agent’ (OR 0.06, p = 0.02) and increased in age group 3 (≥60 years) by hypertension (OR 4.9, p = 0.03) and for macrovascular disease by low HDL cholesterol (OR 3.1, p = 0.03).

**Table 2:** Significant (p<0.05) associations between risk factors and presence or absence of vascular diseases in adult type 1 diabetes.

	Age group (years)		
	20–44	45–59	≥60
**1 microvascular disease**	(n = 141)	(n = 133)	(n = 100)
duration of diabetes (years)	1.10 (1.04,1.17)^<0.001^	1.07 (1.03, 1.11)^<0.001^	1.04 (1.01, 1.07)^0.01^
**2 or more microvascular diseases**	(n = 126)	(n = 109)	(n = 85)
HbA1c	1.03 (1.01, 1.05)^0.004^	ns	ns
duration of diabetes (years)	1.11 (1.02, 1.21)^0.01^	1.12 (1.07, 1.18)^<0.001^	1.05 (1.01, 1.09)^0.01^
hypertension	11.3 (3.42, 37.2)^<0.001^	ns	4.88 (1.12, 21.3)^0.03^
hypercholesterolaemia	ns	0.06 (0.01, 0.68)^0.02^	ns
**Any macrovascular disease**	(n = 145)	(n = 177)	(n = 144)
duration of diabetes	insufficient events	1.07 (1.03, 1.12)^0.002^	1.04 (1.01, 1.07)^0.01^
low HDL cholesterol	insufficient events	ns	3.11 (1.06, 9.13)^0.03^

Independent variables included in each analysis were: HbA1c concentration, diabetes duration, age (within each age category), obesity (BMI ≥30kg/m^2^), hypertension (≥140/90 mmHg, or on blood pressure lowering medication), hypercholesterolaemia (LDL ≥2.5 mmol/L, or on lipid lowering medication), low HDL cholesterol (<1.03 mmol/L) and hypertriglyceridaemia (≥1.7 mmol/L). Odds ratios and 95% confidence intervals are shown with significance levels in superscript. Abbreviation ‘ns’: non-significant.

**Fig 1:**
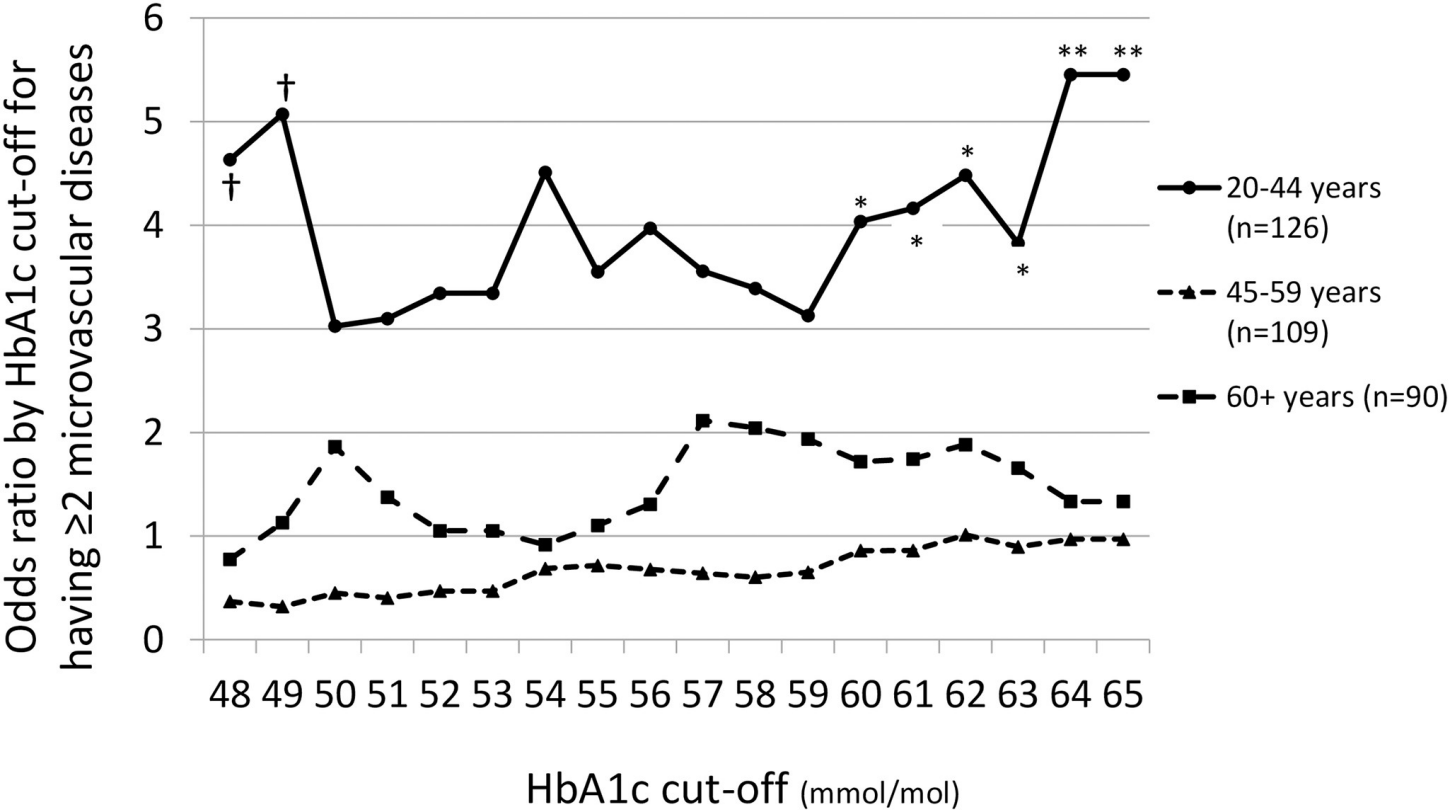
Odds ratios for ≥2 microvascular diseases in those in each of three age categories with an HbA1c greater than or equal to HbA1c cut-offs between 48 and 65 mmol/mol. Significant odds ratios are denoted by: * p<0.05; ** p<0.01. ^†^ 95% confidence intervals for the 48 and 49 mmol/mol HbA1c cut-offs were 0.4–49.5 and 0.5–51.8 mmol/mol, respectively, indicating unreliable odds ratio estimates due to small numbers (<40) in the lower HbA1c range. At higher HbA1c cut-offs, the upper bound of the 95%CI did not exceed 26 mmol/mol.

The increased risk of ≥2 microvascular diseases with increasing HbA1c in the youngest age category was further explored by categorising high HbA1c according to successively higher cut-offs from ≥55 mmol/mol to ≥65 mmol/mol (7.3 to 8.2%). The odds ratio for ≥2 microvascular diseases in those aged 20–44 years was significantly, independently increased by an HbA1c of 60 mmol/mol (7.6%) or more (Fig 1). In those aged 45 years or more there was no significant increase in odds ratio for any categorisation of high HbA1c.

Analyses of variation in the associations between HbA1c and prevalence of vascular disease were similarly carried out using tertiles of duration of diabetes instead of tertiles of age. Duration of diabetes tertiles were: 0–21.80; 31.87–35.99; and 36.06–63.84 years. Associations using categorisation by duration of diabetes were similar to those with categorisation by age but were generally appreciably weaker. There, nevertheless, remained a suggestion of an independent association between HbA1c and having two or more microvascular diseases in the youngest age range, albeit at weaker significance (p = 0.057) than with categorisation by age (p = 0.004). Multivariable logistic analyses were also repeated in women and men separately. The odds ratio (95% confidence interval) and significance for the association between HbA1c and having two or more microvascular diseases was, for women, 1.02 (0.99, 1.05), p = 0.06 and, for men, 1.03 (0.97, 1.10), p = 0.3, indicating no gender effect in relation to our principal finding.

## Discussion

Our hypothesis that greater age would reduce the impact of glycaemia on the risk of vascular complications was supported by our findings, but duration of diabetes was a significant factor in all age ranges. In patients aged ≥45 years HbA1c was not a significant factor contributing towards the prevalence of micro- and macrovascular complications. This implies that intensive blood glucose management in patients aged ≥45 years may have limited benefits in terms of reducing the risk of complications compared with glycaemic control in younger age ranges. However, this does not dismiss the benefits of good glycaemic control in older people with T1DM, nor do our findings argue for excluding blood glucose management in their clinical care.

In the study cohort age group of 20–44 years, the odds ratio for the presence of two, or more microvascular complications became significant in those with an HbA1c of 60 mmol/mol or more. This strengthens the argument for early intensive glucose control, which has been emphasised by the long-term salutary effects of intensive management shown in the EDIC study [3] and the concept of glycaemic tracking [7]. The benefit of optimising glycaemic control in T1DM has been indicated by the Swedish National Diabetes Registry study of Matuleviciene-Anängen et al [9]: In this analysis, risk of acute myocardial infarction was substantially increased in patients with T1D with HbA1c>72mmol/mol and albuminuria but in those with HbA1c<52mmol/mol and no albuminuria risk, ceased to be increased in men and was markedly reduced in women [9]. The duration of follow-up in this study was 20 years, similar to that in the 20–44 year old cohort assessed in our study.

The association between HbA1c and microvascular disease burden in our younger age range is as expected, as hyperglycaemia is the major mechanism for diabetes-related vascular complications [10]. However, no relationship was seen for singular microvascular complications across the range of HbA1c. The possible explanation for this is the high prevalence of retinopathy, which has a low threshold for diagnosis. Many of the cases are likely to be background retinopathy that do not require further treatment [11]. There was a marked increase in prevalence of vascular disease at higher HbA1c levels among young adults, with some carry-over of this effect into middle age (S1 Table). Additionally, this data emphasises the lack of association between vascular disease prevalence and HbA1c level in the elderly.

The absence of macrovascular complications in patients aged 20–44 years with HbA1c <60mmol/mol, can be explained by the duration of diabetes. The median duration of diabetes for this age group was 21 years, shorter than in patients aged 45–59 years and ≥60 years (32 and 37 years of duration respectively).

Inclusion of other metabolic factors in our multivariable analyses was also informative. LDL cholesterol, a known risk factor for macrovascular complication (and observed to significantly increase with increasing HbA1c in the youngest age group—not shown), did not explain the relationship between HbA1c and microvascular complications in those aged 20–44 years. Instead, BP ≥140/90mmHg or anti-hypertensive medications was found to be a prominent factor in this younger age group when predicting microvascular disease.

### Limitations

To fully address our hypothesis, careful, long-term assessment of glycaemia will be required. Moreover, episodes of severe hypoglycaemia, which may affect vascular risk [2,12,13] will need to be evaluated. Nevertheless, it is of interest that, even based on a single assessment of recent glycaemic control using HbA1c, marked differences in the relationships between vascular complications and glycaemia with age were apparent. Glycaemic variability has been shown to adversely affect prognosis of patients, contribute towards the pathogenesis of complications [14] and increase mortality [10], suggesting that this is also an important factor to further investigate. Other risk factors including family history of cardiovascular disease, smoking, alcohol consumption, and physical activity, were not included and may have confounded the results.

## Conclusions

This preliminary study has shown that an increased HbA1c above 60 mmol/mol (7.6%) is associated with increased microvascular complications in patients aged 20–44 years, independent of age and duration of diabetes. This relationship was not apparent in older people with T1DM in whom duration of diabetes was the major factor. Age is closely related to duration of diabetes and, accordingly, our findings emphasise the importance of considering age when establishing personalised HbA1c targets, and ensuring particular attention is given to cardiovascular risks of hypertension and lipids in older people with T1DM. Further investigation of the relationships between age, duration of diabetes and complications in adults with T1DM is warranted.

## Supporting information

S1 Table Distribution of different micro- and macrovascular disease for the specified age groups in relation to HbA1c (mmol/mol). (DOCX)
