# Supplementary material for: Loss of association between HbA1c and vascular disease in older adults with type 1 diabetes
Source: PLoS One. 2020 Jun 15;15(6):e0234319. doi: 10.1371/journal.pone.0234319 (PMC7295188; doi:10.1371/journal.pone.0234319)
Supplement: S1 Table — (DOCX) [file pone.0234319.s001.docx]

**Supplementary Table 1.** Distribution of different micro- and macrovascular disease for the specified age groups in relation to HbA1c (mmol/mol)

|  | Age  20-44y | | | | Age  45-59y | | | | Age  60-89y | | | |  |
| --- | --- | --- | --- | --- | --- | --- | --- | --- | --- | --- | --- | --- | --- |
|  | HbA1c  28-58 | HbA1c  60-69 | HbA1c  70-198 | n  cases | HbA1c  28-58 | HbA1c  60-69 | HbA1c  70-198 | n  cases | HbA1c  28-58 | HbA1c  60-69 | HbA1c  70-198 | n  cases | Total cases |
| Retinopathy | 33% | 21% | 45% | 66 | 27% | 34% | 38% | 99 | 43% | 34% | 23% | 92 | 257 |
| Nephropathy | 25% | 14% | 61% | 28 | 25% | 33% | 42% | 36 | 37% | 35% | 28% | 43 | 107 |
| Neuropathy | 20% | 20% | 60% | 10 | 17% | 30% | 52% | 23 | 31% | 38% | 31% | 29 | 62 |
| Ischaemic heart disease | 0 | 0 | 100% | 1 | 35% | 12% | 53% | 17 | 44% | 47% | 9% | 34 | 52 |
| Peripheral vascular disease | 0 | 0 | 100% | 1 | 21% | 29% | 50% | 14 | 33% | 43% | 24% | 21 | 36 |
| Stroke | 0 | 0 | 0 | 0 | 75% | 25% | 0 | 4 | 33% | 67% | 0 | 9 | 13 |
